# Estimating fMRI Timescale Maps

**DOI:** 10.1101/2025.04.23.650300

**Published:** 2025-05-21

**Authors:** Gabriel Riegner, Samuel Davenport, Bradley Voytek, Armin Schwartzman

**Affiliations:** 1 Halicioğlu Data Science Institute, University of California San Diego; 2 Division of Biostatistics, University of California San Diego; 3 Department of Cognitive Science, University of California San Diego; 4 Neurosciences Graduate Program, University of California San Diego

**Keywords:** time-domain linear model, autocorrelation-domain nonlinear model, uncertainty quantification, statistical inference, human connectome project, functional brain organization

## Abstract

Brain activity unfolds over hierarchical timescales that reflect how brain regions integrate and process information, linking functional and structural organization. While timescale studies are prevalent, existing estimation methods rely on the restrictive assumption of exponentially decaying autocorrelation and only provide point estimates without standard errors, limiting statistical inference. In this paper, we formalize and evaluate two methods for mapping timescales in resting-state fMRI: a time-domain fit of an autoregressive (AR1) model and an autocorrelation-domain fit of an exponential decay model. Rather than assuming exponential autocorrelation decay, we define timescales by projecting the fMRI time series onto these approximating models, requiring only stationarity and mixing conditions while incorporating robust standard errors to account for model misspecification. We introduce theoretical properties of timescale estimators and show parameter recovery in realistic simulations, as well as applications to fMRI from the Human Connectome Project. Comparatively, the time-domain method produces more accurate estimates under model misspecification, remains computationally efficient for high-dimensional fMRI data, and yields maps aligned with known functional brain organization. In this work we show valid statistical inference on fMRI timescale maps, and provide Python implementations of all methods.

## 1 Introduction

### 1.1 fMRI Timescale Maps

Neural processes span multiple timescales, from millisecond synaptic events to slower activity coordinating distributed brain networks (Buzsáki, 2004). Multimodal evidence links these differences in timescales to intrinsic brain organization that reveal how regions integrate and process information over time. Timescale maps of the brain align with functional hierarchy – sensory areas that process rapidly changing stimuli show faster timescales than association areas involved in cognitive processes that unfold over longer durations (Raut et al., 2020; Gao et al., 2020; Hasson et al., 2008; Murray et al., 2014; Stephens et al., 2013). This hierarchy is also associated with anatomical organization including myelination levels and gene expression patterns, as shown in studies using human electrophysiology, MEG, and gene expression profiling (Gao et al., 2020; Shafiei et al., 2023).

Computational modeling by Li and Wang (2022) suggests that hierarchical timescales emerge from: (1) brain-wide gradients in synaptic excitation strength, (2) electrophysiological differences between excitatory and inhibitory neurons, and (3) balance between distant excitatory and local inhibitory inputs. In addition to these intrinsic mechanisms, there is growing evidence that neuronal timescales are dynamic and modulated by experimental manipulations or behavioral demands. For example, pharmacological agents like propofol and serotonergic drugs alter intrinsic timescales, affecting the temporal integration of information in the brain (Huang et al., 2018; Shinn et al., 2023). Timescale changes have also been observed during development, sleep deprivation, wakefulness, neuropsychiatric disorders (autism and schizophrenia), and naturalistic behaviors (Martin-Burgos et al., 2024; Meisel et al., 2017; Watanabe et al., 2019; Wengler et al., 2020; Manea et al., 2024). These findings demonstrate that timescales are broadly relevant to both structural and functional properties of the brain.

Seminal research on timescales has primarily used invasive electrophysiology in non-human animals (Murray et al., 2014; Cirillo et al., 2018; Nougaret et al., 2021; Manea et al., 2022; Spitmaan et al., 2020; Trepka et al., 2024). While these methods provide high temporal resolution for studying neural activity at the single-neuron level, they are limited by sparse spatial sampling. Investigating the large-scale spatial organization of timescale maps requires non-invasive methods like resting-state functional MRI (rfMRI), which measures spontaneous fluctuations in the blood oxygen level-dependent (BOLD) signal. rfMRI provides full-brain coverage of hemodynamic processes at frequencies below 0.1 Hz (Raut et al., 2020; He, 2011), offering dense spatial sampling compared to techniques like EEG or MEG. Although the BOLD signal does not directly measure neural activity, it reflects hemodynamic changes associated with underlying electrophysiological signals (Logothetis, 2008), making it a valuable tool for investigating high-resolution cortical timescale maps. Studies have shown that rfMRI-derived timescale maps align spatially with those from other imaging modalities across human and animal models (Raut et al., 2020; Shafiei et al., 2020; Lurie et al., 2024). The present study will focus on rfMRI from the Human Connectome Project dataset (Van Essen et al., 2013).

### 1.2 Current methods

Timescales are generally estimated using three main methods in the (1) time-domain, (2) autocorrelation-domain, or (3) frequency-domain. The most common is the autocorrelation domain, where timescales are defined by fitting an exponential decay model to the sample autocorrelation function (ACF) (Rossi-Pool et al., 2021; Cirillo et al., 2018; Ito et al., 2020; Runyan et al., 2017; Zeraati et al., 2022; Nougaret et al., 2021; Wasmuht et al., 2018; Müller et al., 2020; Maisson et al., 2021; Li and Wang, 2022; Shafiei et al., 2020). Similar approaches compute timescales directly from the sample ACF as the sum of positive autocorrelations (Wengler et al., 2020; Manea et al., 2022; Watanabe et al., 2019), or by identifying where the sample ACF crosses a specified threshold (Wengler et al., 2020; Zilio et al., 2021). Alternatively, the time-domain method uses a first-order autoregressive (AR1) model to estimate timescales directly from time-series data (Kaneoke et al., 2012; Meisel et al., 2017; Huang et al., 2018; Lurie et al., 2024; Shinn et al., 2023; Shafiei et al., 2020; Spitmaan et al., 2020; Trepka et al., 2024), and has shown better test-retest reliability than autocorrelation-domain methods for rfMRI (Huang et al., 2018). Finally, frequency-domain methods use the sample power-spectral density (PSD) to estimate and remove neural oscillations, as timescales are properties of the aperiodic signal (Donoghue et al., 2020; Gao et al., 2020; Manea et al., 2024; Zeraati et al., 2022; Fallon et al., 2020). Since previous research shows that rfMRI is predominantly aperiodic with scale-free spectral properties (He et al., 2010; He, 2011), the present paper focuses only on the time- and autocorrelation-domain methods.

### 1.3 Problem Statement and Proposed Solution

A key challenge in applied timescale research is the lack of standardized model definitions, where diverse approaches have led to inconsistent findings across studies (Zeraati et al., 2022; Fallon et al., 2020; Shafiei et al., 2020). Many parameterization methods rely on restrictive assumptions, such as exponential autocorrelation decay, which may bias timescale estimates and (more often) their standard errors (Woolrich et al., 2001; Zeraati et al., 2022; Raut et al., 2019). Additionally, the distributional properties of these methods are often ignored, resulting in studies reporting only point estimates without quantifying uncertainty, which limits statistical inference and hypothesis testing (Newey and West, 1987; White and Domowitz, 1984).

To address these issues, this paper formalizes and evaluates two commonly applied timescale methods: the time-domain fit of an autoregressive (AR1) model and the autocorrelation-domain fit of an exponential decay model in rfMRI. The goal is to estimate accurate timescale maps that enable robust statistical testing and inference across brain regions. This work offers the following contributions: (1) The assumptions are generalized to include all stationary and mixing processes, not only those with exponential autocorrelation decay. (2) Robust standard errors account for the inevitable mismatch between the data-generating process and fitted model, enabling valid inference despite model misspecification. (3) Theoretical properties demonstrate that both time- and autocorrelation-domain estimators converge to different values due to their distinct definitions, and are consistent and asymptotically normal. (4) Simulations confirm that both methods yield unbiased estimates across autoregressive and realistic settings, with standard errors that are robust to non-exponential autocorrelation decay. (5) Empirical analysis of rfMRI from the Human Connectome Project shows that both approaches yield similar t-ratio maps (timescales relative to their standard errors), revealing a hierarchical organization of timescales across the cortex. While this hierarchy aligns with prior point estimate maps, our approach improves interpretability by accounting for variability, ensuring that observed patterns are not artifacts of sampling noise or model misspecification. (6) Comparative analyses show that the time-domain method performs as well as, and often better than, the autocorrelation-domain method, while maintaining greater computational efficiency for high-dimensional fMRI data analysis.

The proposed methods address important limitations in neural timescale research by providing rigorous statistical methods that move beyond point estimates to incorporate uncertainty quantification. Through formal definitions, theoretical validation, and extensive testing across simulations and empirical data, we demonstrate that both time- and autocorrelation-domain estimators yield consistent standard errors under broad conditions, enabling reliable inference and hypothesis testing. This work establishes a methodological foundation for future research investigating the functional and structural organization of timescales in the brain.

## 2 Methods

### 2.1 Assumptions

Let $\left\{X_{t},t\in Z\right\}$ be a discrete-time stochastic process that is *weakly stationary* and *strong mixing*, and let $x_{t}=\left\{x_{1},x_{2},\ldots ,x_{T}\right\}$ be an observed sample of $X_{t}$. For simplicity, assume $X_{t}$ and $x_{t}$ have mean zero. Stationarity implies a constant mean and variance (independent of time index t), and an autocovariance function that only depends on time lag k:

(1) $$\gamma_{k}=cov\left[X_{t},X_{t-k}\right]=E\left[X_{t}X_{t-k}\right].$$

For analysis, we use a normalized measure of the autocovariances, the *autocorrelation function (ACF)*:

(2) $$\rho_{k}=corr\left(X_{t},X_{t-k}\right)=\gamma_{0}^{-1}\gamma_{k},$$

where $\gamma_{k}$ is the autocovariance at lag k and $\gamma_{0}$ is the variance. Cauchy-Schwarz bounds $\left|\rho_{k}\right|\leq 1$, but stationarity alone does not guarantee decay of $\rho_{k}$ with increasing lag; constant or periodic processes can maintain nonzero correlations indefinitely. Strong mixing (α-mixing) imposes stronger dependence constraints than stationarity while still allowing for a wide set of stochastic processes. By definition, a process is strong mixing if α(ℓ)→0 as ℓ→∞, where α(ℓ) measures the maximum dependence between events separated by ℓ time points. Strong mixing implies ergodicity (Hansen, 2022, Chapter 14.12), which ensures consistent estimation by the ergodic theorem (Hansen, 2022, Theorem 14.9). Additionally, if a mixing process has r>2 finite moments $E{\left|X_{t}\right|}^{r}<\infty$ and its mixing coefficients satisfy $\sum_{\ell =0}^{\infty }\alpha {\left(\ell \right)}^{1-2/r}<\infty$, then its autocorrelations decay sufficiently fast for application of the central limit theorem for dependent data (Hansen, 2022, Theorem 14.15). These conditions justify defining a timescale as the rate of autocorrelation decay, and support asymptotic theory for estimation and inference on timescale maps (see Estimator Properties).

As introduced by Murray et al. (2014), the timescale τ represents the lag where exponentially decaying autocorrelations reach 1/e≈0.37 (e-folding time), analogous to the time constants of many physical systems. While it provides an intuitive description of the memory or persistence of that process, assuming an exponential function imposes stricter constraints than strong mixing, which alone does not prescribe any specific type of decay (exponential, linear, damped periodic, etc.). This highlights an important distinction between the data-generating process and the simplified parametric model used to describe the timescale at which such a process becomes decorrelated. In the present paper, we adopt broad assumptions, requiring only that the process is stationary and mixing, to account for cases where the ACF decay may not be strictly exponential. Acknowledging that the data-generating process and the fitted model will likely be different in practice, we describe standard error estimation methods that account for this mismatch, enabling valid inference despite model misspecification.

### 2.2 Timescale Definitions

We approximate the dominant exponential decay in autocorrelations by a single timescale parameter τ, and formally evaluate two timescale methods that are commonly applied across neuroimaging modalities (fMRI, EEG, ECoG, MEG). The time-domain linear model estimated with linear least squares (Kaneoke et al., 2012; Meisel et al., 2017; Huang et al., 2018; Lurie et al., 2024; Shinn et al., 2023; Shafiei et al., 2020; Spitmaan et al., 2020; Trepka et al., 2024), and the autocorrelation-domain nonlinear model estimated with nonlinear least squares (Rossi-Pool et al., 2021; Cirillo et al., 2018; Ito et al., 2020; Runyan et al., 2017; Zeraati et al., 2022; Nougaret et al., 2021; Wasmuht et al., 2018; Müller et al., 2020; Maisson et al., 2021; Li and Wang, 2022; Shafiei et al., 2020).

#### 2.2.1 Time-Domain Linear Model

A first order autoregressive model (AR1) provides a linear approximation of timescale. The AR1 model:

(3) $$X_{t}=\phi X_{t-1}+e_{t},$$

models the process as a linear regression between $X_{t}$ and $X_{t-1}$ in the time domain with *iid* errors. In the autocorrelation domain, it implies that the theoretical ACF decays exponentially at a rate determined by ϕ, such that $\rho_{k}=\phi^{k}$ (Hansen, 2022, Chapter 14.22). For a stationary process with |ϕ|<1, the exponential decay rate can be directly obtained from ϕ, with a timescale τ equal to the lag at which the AR1-projected ACF reaches 1/e≈0.37, that is, $\rho_{\tau }=\phi^{\tau }=1/e$, resulting in τ=g(ϕ)=-1/log(|ϕ|). The timescale τ is expressed as a nonlinear function of ϕ, denoted by g(ϕ). This defines τ to be a real number even though the ACF only includes integer indices, and the absolute value allows for ϕ<0.

Importantly, we do not assume that the observed process $X_{t}$ actually follows the AR1 model from equation (3). This allows for projections errors that may exhibit unequal variance and residual autocorrelation. Relaxing the constraints on the errors allows for AR1 approximations in which deviations from AR1 are captured by the error term. Thus, this model can be applied to any stationary and mixing process, even if the true data-generating process is not AR1, making the resulting fit an AR1 projection. The parameter $\phi^{*}$ then represents the best approximation of the process $X_{t}$ by an AR1 model. It is the value that minimizes the expected squared error function S(ϕ):

(4) $$S(\phi )=E[{\left(X_{t}-\phi X_{t-1}\right)}^{2}],\;{\;\phi }^{*}=\underset{\phi }{argmin\;}S(\phi ).$$

S(ϕ) is minimized by taking its derivative with respect to ϕ, setting it to zero, and solving for $\phi^{*}$:

(5) $$\frac{d}{d\phi }S(\phi )=-2E\left[X_{t-1}\left(X_{t}-\phi X_{t-1}\right)\right]=0.$$

Differentiating this quadratic function yields a linear equation in ϕ, and solving this results in a closed-form expression for the optimal $\phi^{*}$. Therefore, $\phi^{*}$ is defined by *linear projection* and the timescale parameter $\tau^{*}$ by a change of variable:

(6) $$\phi^{*}={\left(E\left[X_{t-1}^{2}\right]\right)}^{-1}\left(E\left[X_{t}X_{t-1}\right]\right),$$

(7) $$\tau^{*}=g\left(\phi^{*}\right)=-\frac{1}{\log\left(\left|\phi^{*}\right|\right)}.$$

In other words, the timescale parameter $\tau^{*}$ represents the timescale (1/e autocorrelation decay) of the best AR1 approximation of the observed process. Since $X_{t}$ is stationary with finite variance, the parameters $\phi^{*}$ and $\tau^{*}$ defined by projection are unique; in fact, any approximating AR1 model is identifiable if $E\left[X_{t-1}^{2}\right]$ is non-negative (Hansen, 2022, Theorem 14.28).

#### 2.2.2 Autocorrelation-Domain Nonlinear Model

Alternatively, timescales can be defined in the autocorrelation domain by an exponential decay function, as introduced by Murray et al. (2014). For consistent notation, we write the autocorrelation-domain nonlinear model as:

(8) $$\rho_{k}=\phi^{k}+e_{k},\;\text{for}\;k\in \{0,\;1,\ldots ,K\},$$

where $\rho_{k}$ denotes the autocorrelation at lag k and $e_{k}$ is the error term. The relationship between $\rho_{k}$ and k is nonlinear in ϕ which determines the exponential decay rate. Unlike the Time-Domain Linear Model above, this definition captures exponential decay across multiple (K) lags of the ACF rather than by a single lag, capturing longer-range temporal dependencies. Consequently, the parameter ϕ here is not the same as the AR1 projection parameter, since both its value and interpretation differ when influenced by multiple lags.

Here, the projection parameter $\phi^{*}$ is the value that minimizes the expected squared error function S(ϕ):

(9) $$S(\phi )=E[{\left(\rho_{k}-\phi^{k}\right)}^{2}],\;\phi^{*}=\underset{\phi }{argmin\;}S(\phi ).$$

S(ϕ) is minimized by taking its derivative with respect to ϕ, setting it to zero, and solving for ϕ:

(10) $$\frac{d}{d\phi }S(\phi )=-2E[\left(k\phi^{k-1})(\rho_{k}-\phi^{k}\right)]=0.$$

However, the derivative is nonlinear in ϕ, preventing a closed-form solution for least squares minimization. Therefore, optimization methods are needed to approximate $\phi^{*}$ by *nonlinear projection*. Like before, the corresponding timescale is defined as the time lag at which the fitted ACF reaches 1/e and can be expressed by the change or variable:

(11) $$\tau^{*}=g\left(\phi^{*}\right)=-\frac{1}{\log\left(\left|\phi^{*}\right|\right)}.$$

### 2.3 Timescale Estimation

#### 2.3.1 Time-Domain Linear Least Squares Estimator

Given observations $x_{1},\ldots ,x_{T}$, the linear least squares (LLS) estimator of the Time-Domain Linear Model is obtained by replacing the expectations in equation (6). It has the following closed-form expression:

(12) $${\overset{ˆ}{\phi }}_{LLS}={\left(\sum_{t=2}^{T}x_{t-1}^{2}\right)}^{-1}\left(\sum_{t=2}^{T}x_{t}x_{t-1}\right),$$

(13) $${\overset{ˆ}{\tau }}_{LLS}=g({\overset{ˆ}{\phi }}_{LLS})=-\frac{1}{log(\left|{\overset{ˆ}{\phi }}_{LLS}\right|)},$$

where ${\overset{ˆ}{\phi }}_{\text{LLS}}$ and ${\overset{ˆ}{\tau }}_{\text{LLS}}$ are the sample versions of the population parameters from equations (6) and (7), respectively (Hansen, 2022, Chapter 14.3).

#### 2.3.2 Autocorrelation-Domain Nonlinear Least Squares Estimator

The nonlinear least squares (NLS) estimator of the Autocorrelation-Domain Nonlinear Model is fit to the ACF, so the time series needs to be first transformed into the autocorrelation domain. For a finite and centered time series, the population ACF from equation (2) is estimated by:

(14) $${\overset{ˆ}{\rho }}_{k}={\overset{ˆ}{\gamma }}_{0}^{-1}{\overset{ˆ}{\gamma }}_{k}={\left(\sum_{t=1}^{T}x_{t}^{2}\right)}^{-1}\left(\sum_{t=k+1}^{T}x_{t}x_{t-k}\right),$$

where ${\overset{ˆ}{\gamma }}_{k}$ is the sample covariance at lag k and ${\overset{ˆ}{\gamma }}_{0}$ is the sample variance. The population ACF (2) by mixing approaches zero as lag k increases. However, sampling variability may yield non-zero autocorrelations even when true values are zero. To mitigate this, the sample ACF estimator (14) imposes a bias towards zero by scaling the autocovariances (${\overset{ˆ}{\gamma }}_{k}$, calculated using T-k terms) by the total sample variance (${\overset{ˆ}{\gamma }}_{0}$, calculated using all T timepoints).

By the model definition (8), the exponential decay parameter $\phi^{*}$ that minimizes the cost function, S(ϕ) in equation (9), is estimated by minimizing the sample analog $\hat{S}(\phi )$:

(15) $$\hat{S}\left(\phi \right)=\frac{1}{K}\sum_{k=0}^{K}{\left({\overset{ˆ}{\rho }}_{k}-\phi^{k}\right)}^{2},$$

(16) $${\overset{ˆ}{\phi }}_{NLS}^{*}=\underset{\phi }{argmin\;}\hat{S}\left(\phi \right),$$

(17) $${\overset{ˆ}{\tau }}_{NLS}^{*}=g({\overset{ˆ}{\phi }}_{NLS}^{*})=-\frac{1}{log(\left|{\overset{ˆ}{\phi }}_{NLS}\right|)}.$$

In this paper we use the Levenberg-Marquart algorithm to iteratively update the estimate of ${\overset{ˆ}{\phi }}_{NLS}^{*}$ until convergence (i.e., when the step size goes below a 10^−6^ tolerance).

### 2.4 Standard Error of the Estimators

#### 2.4.1 Time-Domain Method

We provide a standard error expression for $\tau^{*}$ under model misspecification. When the data-generating process is not AR1 and consequently the errors are not independent, the usual (naive) standard errors will have a downward bias. This renders invalid confidence intervals or hypothesis tests that rely on them. To correct for this, the Newey and West (1987) (NW) expression takes a sandwich form and explicitly accounts for misspecification by summing the covariance structure of the errors, ensuring that the resulting standard errors are asymptotically valid (Hansen, 2022, Theorem 14.32).

Given that $X_{t}$ is stationary and mixing, so too are the errors from equation (3) since these properties are preserved by finite transformations (Hansen, 2022, Theorem 14.2 and Theorem 14.12). Consequently, the autocovariances of the errors vanish as the time lag increases (see Assumptions). Further, because the timescale $\tau^{*}$ is given by the nonlinear function $g\left(\phi^{*}\right)$ (see equation (7)) with derivative $\frac{d}{d\phi }g\left(\phi^{*}\right)$, its standard error can be approximated using the delta method:

(18) $${se}_{NW}\left(\phi^{*}\right)=\sqrt{q^{-1}\omega q^{-1}},\;{se}_{NW}\left(\tau^{*}\right)\approx {se}_{NW}\left(\phi^{*}\right)\cdot \frac{d}{d\phi }g\left(\phi^{*}\right),$$

where

(19) $$q=E\left[X_{t-1}^{2}\right]\;\text{and}\;\omega =\sum_{\ell =-\infty }^{\infty }E\left[\left(X_{t-1}\cdot e_{t}\right)\left(X_{t-1-\ell }\cdot e_{t-\ell }\right)\right].$$

The covariance terms in ω capture deviations in the error structure from the standard *iid* case. For the special case of correct specification, when $X_{t}$ is a true AR1 process, the standard error of the AR1 coefficient $\phi^{*}$ reduces to the usual formula:

(20) $${se}_{\text{Naive}\;}\left(\phi^{*}\right)=\sqrt{\sigma^{2}q^{-1}},$$

where $\sigma^{2}$ is the error variance.

#### 2.4.2 Autocorrelation-Domain Method

Equation (8) models autocorrelation decay using a parametric exponential function. While usual (naive) applications assume *iid* errors, our approach permits dependent errors generated by an underlying stationary and mixing process. The standard errors proposed below account for potential misspecification of the exponential form and are asymptotically valid under these more general conditions.

Following the description in Hansen (2022, Chapter 22.8 and Chapter 23.5), if $\phi^{*}$ uniquely minimizes S(ϕ) in equation (9), such that $S(\phi )>S\left(\phi^{*}\right)$ for all $\phi \neq \phi^{*}$, the precision of $\phi^{*}$ can be computed using a Newey and West (1987) (NW) form that reflects both the curvature of the squared loss function at its minimum and the covariance of the errors. Further, because the timescale $\tau^{*}$ is given by the nonlinear function $g\left(\phi^{*}\right)$ with derivative $\frac{d}{d\phi }g\left(\phi^{*}\right)$, its standard error can be approximated by the delta method:

(21) $${se}_{NW}\left(\phi^{*}\right)=\sqrt{q^{-1}\omega q^{-1}},\;{se}_{NW}\left(\tau^{*}\right)\approx {se}_{NW}\left(\phi^{*}\right)\cdot \frac{d}{d\phi }g\left(\phi^{*}\right).$$

The components q and ω are derived from the regression function $m(k,\phi )=\phi^{k}$ in (8) and its derivative $m_{\phi ,k}=\frac{d}{d\phi }m(k,\phi )=k\phi^{k-1}$, defined as:

(22) $$q=E[m_{\phi^{*},k}^{2}]=E[{\left(k\phi^{*k-1}\right)}^{2}]\;\text{and}\;\omega =\sum_{\ell =-\infty }^{\infty }E[\left(m_{\phi^{*},k}\cdot e_{k})(m_{\phi^{*},k-\ell }\cdot e_{k-\ell }\right)].$$

The derivative of the regression function $m_{\phi^{*},k}$ evaluated at $\phi^{*}$ locally approximates the nonlinear model by a linear one, and the expression for ω sums the covariance structure of the errors, ensuring that the standard errors are asymptotically valid even with model deviations (see (42)). This is a realistic scenario under the mild conditions of stationarity and mixing. In the special case where the errors are *iid*, the standard error of $\phi^{*}$ simplifies to the usual formula:

(23) $${se}_{\text{Naive}\;}\left(\phi^{*}\right)=\sqrt{\sigma^{2}q^{-1}},$$

where $\sigma^{2}$ is the error variance.

#### 2.4.3 Autocorrelation/Time-Domain Method

As discussed, ϕ defined in the autocorrelation domain by nonlinear projection (9) captures longer range autocorrelations than when it is defined in the time domain (6). However, it assumes a signal + noise form for the ACF $\left[\rho_{k}=\phi^{k}+e_{k}\;(8)\right]$ which is not realistic for many stochastic processes. For example, a correctly specified AR1 process has an ACF with no additive error ($\rho_{k}=\phi^{k}$). For approximating higher order autoregressive processes, the deviations $e_{k}$ represent misspecification error – the part of the ACF that the approximating model fails to explain. In many cases this error might be systematic and not random, and therefore the definition of standard error from (21) would be incorrect. To address this problem, we propose a hybrid approach where the timescale is defined in the autocorrelation domain by (9) but its standard error is defined in the time domain by (18).

### 2.5 Standard Error Estimation

#### 2.5.1 Time-Domain Method

The sample standard error estimator takes the form:

(24) $${\hat{se}}_{NW}({\overset{ˆ}{\phi }}_{LLS}^{*})=\sqrt{{\overset{ˆ}{q}}^{-1}\overset{ˆ}{\omega }{\overset{ˆ}{q}}^{-1}},\;{\hat{se}}_{NW}({\overset{ˆ}{\tau }}_{LLS}^{*})\approx {\hat{se}}_{NW}({\overset{ˆ}{\phi }}_{LLS}^{*})\cdot \frac{d}{d\phi }g({\overset{ˆ}{\phi }}_{LLS}^{*})$$

where

(25) $$\overset{ˆ}{q}=\frac{1}{T}\sum_{t=2}^{T}x_{t-1}^{2}\;\text{and}\;\overset{ˆ}{\omega }=\sum_{\ell =-M}^{M}\left(1-\frac{\left|\ell \right|}{M\;+\;1}\right)\;\frac{1}{T}\sum_{1\leq t-\ell \leq T}\left(x_{t-1}\cdot {\overset{ˆ}{e}}_{t}\right)\left(x_{t-1-\ell }\cdot {\overset{ˆ}{e}}_{t-\ell }\right).$$

This estimator calculates a weighted sum of the regression scores $x_{t-1}\cdot {\overset{ˆ}{e}}_{t}$, where ${\overset{ˆ}{e}}_{t}=x_{t}-{\overset{ˆ}{\phi }}_{LLS}^{*}\cdot x_{t-1}$. The true ω is approximated by $\overset{ˆ}{\omega }$ by taking a finite sum of the regression score covariances up to lag M, where M is the lag-truncation (or bandwidth). The weights used in the sum decrease linearly with lag ℓ, following a Bartlett kernel (Newey and West, 1987). This kernel not only ensures the standard errors remain non-negative but also regularizes $\overset{ˆ}{\omega }$ to change smoothly with M (Hansen, 2022, Chapter 14.35).

For comparison we also include the naive estimator which simplifies under correct specification:

(26) $${\hat{se}}_{\text{Naive}}({\overset{ˆ}{\phi }}_{LLS})=\sqrt{{\overset{ˆ}{\sigma }}^{2}{\overset{ˆ}{q}}^{-1}},$$

(27) $${\hat{se}}_{\text{Naive}\;}({\overset{ˆ}{\tau }}_{LLS})\approx {\hat{se}}_{\text{Naive}\;}({\overset{ˆ}{\phi }}_{LLS})\frac{d}{d\phi }g({\overset{ˆ}{\phi }}_{LLS}),$$

where ${\overset{ˆ}{\sigma }}^{2}=1/T\sum_{t=2}^{T}{\overset{ˆ}{e}}_{t}^{2}$ is an estimate of the error variance.

#### 2.5.2 Autocorrelation-Domain Method

The sample standard error estimator takes the form:

(28) $${\hat{se}}_{NW}({\overset{ˆ}{\phi }}_{NLS}^{*})=\sqrt{{\overset{ˆ}{q}}^{-1}\overset{ˆ}{\omega }{\overset{ˆ}{q}}^{-1}},\;{\hat{se}}_{NW}({\overset{ˆ}{\tau }}_{NLS}^{*})\approx {\hat{se}}_{NW}({\overset{ˆ}{\phi }}_{NLS}^{*})\cdot \frac{d}{d\phi }g({\overset{ˆ}{\phi }}_{NLS}^{*}),$$

where

(29) $$\overset{ˆ}{q}=\frac{1}{K}\sum_{k=0}^{K}{\left({\overset{ˆ}{m}}_{\phi ,k}\right)}^{2}=\frac{1}{K}\sum_{k=0}^{K}{\left(k{\overset{ˆ}{\phi }}_{NLS}^{*k-1}\right)}^{2},$$

(30) $$\overset{ˆ}{\omega }=\sum_{\ell =-M}^{M}\left(1-\frac{\left|\ell \right|}{M\;+\;1}\right)\;\frac{1}{K}\sum_{1\leq k-\ell \leq K}\left({\overset{ˆ}{m}}_{\phi ,k}\cdot {\overset{ˆ}{e}}_{k}\right)\left({\overset{ˆ}{m}}_{\phi ,k-\ell }\cdot {\overset{ˆ}{e}}_{k-\ell }\right).$$

This estimator calculates a weighted sum of the linearized regression scores ${\overset{ˆ}{m}}_{\phi ,k}\cdot {\overset{ˆ}{e}}_{k}$, where ${\overset{ˆ}{e}}_{k}={\overset{ˆ}{\rho }}_{k}-{\left({\overset{ˆ}{\phi }}_{NLS}^{*}\right)}^{k}$. The estimate of $\overset{ˆ}{\omega }$ takes a finite sum of these scores up to lag M, weighted by a Bartlett kernel, so that $\overset{ˆ}{\omega }$ changes smoothly with M.

In the case of correct specification the equation simplifies to:

(31) $${\hat{se}}_{\text{Naive}\;}({\overset{ˆ}{\phi }}_{NLS}^{*})=\sqrt{{\overset{ˆ}{\sigma }}^{2}{\overset{ˆ}{q}}^{-1}},$$

(32) $${\hat{se}}_{\text{Naive}\;}({\overset{ˆ}{\tau }}_{NLS}^{*})\approx {\hat{se}}_{\text{Naive}\;}({\overset{ˆ}{\phi }}_{NLS}^{*})\frac{d}{d\phi }g({\overset{ˆ}{\phi }}_{NLS}^{*}),$$

where ${\overset{ˆ}{\sigma }}^{2}=1/K\sum_{k=0}^{K}{\overset{ˆ}{e}}_{k}^{2}$ is an estimate of the error variance.

#### 2.5.3 Autocorrelation/Time-Domain Method

The sample standard error for the hybrid method is equivalent to (24), except that the LLS estimator ${\overset{ˆ}{\phi }}_{\text{LLS}}^{*}$ is replaced with the NLS estimator ${\overset{ˆ}{\phi }}_{\text{NLS}}^{*}$, which redefines the errors to be in the time domain ${\overset{ˆ}{e}}_{t}=x_{t}-{\overset{ˆ}{\phi }}_{NLS}^{*}\cdot x_{t-1}$.

### 2.6 Estimator Properties

In this section, we describe the large-sample properties of both the Time-Domain Linear Model and Autocorrelation-Domain Nonlinear Model, focusing on the consistency and limiting variance of their respective estimators. Under general conditions — when the time-domain method is applied to a process that is not AR(1), or the autocorrelation-domain method is applied to a decay process that is not exponential — we demonstrate that the asymptotic distribution is Gaussian, with a limiting variance that can be consistently estimated. Consequently, the resulting t-ratios (see equation (54)) are also asymptotically Gaussian. This allows for the construction of hypothesis tests and confidence intervals across timescale maps of the brain.

#### 2.6.1 Time-Domain Method

Following the description in Hansen (2022, Theorem 14.29), the ergodic theorem shows that mixing (which implies ergodicity) is a sufficient condition for *consistent estimation*. Since $X_{t}$ is stationary and ergodic, so too are $X_{t}X_{t-1}$ and $X_{t-1}^{2}$, and as T→∞:

(33) $$\frac{1}{T}\sum_{t=2}^{T}x_{t}x_{t-1}\;\underset{p}{\to }E\left[X_{t}X_{t-1}\right],$$

(34) $$\frac{1}{T}\sum_{t=2}^{T}x_{t-1}^{2}\underset{p}{\to }E[X_{t-1}^{2}].$$

Applying the continuous mapping theorem yields:

(35) $${\overset{ˆ}{\phi }}_{LLS}^{*}={\left(\frac{1}{T}\sum_{t=2}^{T}x_{t-1}^{2}\right)}^{-1}\left(\frac{1}{T}\sum_{t=2}^{T}x_{t}x_{t-1}\right)\underset{p}{\to }E{\left[X_{t-1}^{2}\right]}^{-1}E\left[X_{t}X_{t-1}\right]=\phi^{*}.$$

This shows that the coefficients of the Time-Domain Linear Model can be consistently estimated by least squares, for any stationary and mixing process with parameters defined by projection in equation (6). Similarly for the regression score estimator in equation (25):

(36) $$\overset{ˆ}{\omega }\underset{p}{\to }\omega .$$

Following Hansen (2022, Theorem 14.33), the asymptotic distribution under general dependence states that the *limiting variance* of ϕ can be approximated using a central limit theorem for correlated observations. With the sample standard errors define in equation (24), as T→∞:

(37) $$\frac{{\overset{ˆ}{\phi }}_{LLS}^{*}\;-\;\phi^{*}}{{\hat{Se}}_{NW}({\overset{ˆ}{\phi }}_{LLS}^{*})}\underset{d}{\to }𝒩(0,\;1).$$

And by the delta method we obtain the limiting variance for the timescale τ, for the denominator defined in (24):

(38) $$\frac{{\overset{ˆ}{\tau }}_{LLS}^{*}\;-\;\tau^{*}}{{\hat{Se}}_{NW}({\overset{ˆ}{\tau }}_{LLS}^{*})}\underset{d}{\to }𝒩(0,\;1).$$

#### 2.6.2 Autocorrelation-Domain Method

To show *consistent estimation*, unlike the time-domain method above where we apply the ergodic theorem to the explicit closed-form expression of the estimator, this is not possible for nonlinear estimators because there is no algebraic expression. Instead, nonlinear least squares minimizes the sample objective function $\hat{S}(\phi )$ from equation (15), which is itself a sample average. By Hansen (2022, Theorem 22.1), for any ϕ, the weak law of large numbers shows that:

(39) $$\hat{S}\left(\phi \right)\underset{p}{\to }S\left(\phi \right).$$

Further, if the minimizer $\phi^{*}$ is unique, $S(\phi )>S(\phi^{*})$ for all $\phi \neq \phi^{*}$, then the sample minimizer from equation (16) converges in probability to the true minimum as K→∞:

(40) $${\overset{ˆ}{\phi }}_{NLS}^{*}\underset{p}{\to }\phi^{*}.$$

This shows that the parameters of the Autocorrelation-Domain Nonlinear Model can be consistently estimated by least squares. Similarly for the regression score estimator in equation (30):

(41) $$\overset{ˆ}{\omega }\underset{p}{\to }\omega .$$

With the additional assumption that the objective function (9) is Lipschitz-continuous for ϕ near $\phi^{*}$, following Hansen (2022, Theorem 23.2), we can approximate the *limiting variance* of $\phi^{*}$ and $\tau^{*}$ using a central limit theorem for correlated observations. Under general conditions, the nonlinear least squares estimator has an asymptotic distribution with a similar structure to that of the linear least squares estimator above; it converges to a Gaussian distribution with a sandwhich-form variance. With the sample standard errors define in (28), as K→∞:

(42) $$\frac{{\overset{ˆ}{\phi }}_{NLS}^{*}\;-\;\phi^{*}}{{\hat{Se}}_{NW}({\overset{ˆ}{\phi }}_{NLS}^{*})}\underset{d}{\to }𝒩(0,\;1).$$

And by the delta method we obtain the limiting variance for the timescale $\tau^{*}$, for the denominator defined in (28):

(43) $$\frac{\overset{ˆ}{\tau^{*}}\;-\;\tau^{*}}{{\hat{Se}}_{NW}({\overset{ˆ}{\tau^{*}}}_{NLS})}\underset{d}{\to }𝒩(0,\;1).$$

## 3 Simulations

### 3.1 Simulation Settings

Monte Carlo simulations with N=10,000 replications were used to evaluate time- and autocorrelation-domain methods. Time series realizations $x_{t}=\left\{x_{1},x_{2},\ldots ,x_{T}\right\}$ with T=4800 were based on three data-generating processes, each characterized by a different autocorrelation structure: AR1, AR2, and autocorrelations derived from rfMRI data. All autocorrelation structures shared the same AR1 projection ($\phi_{\text{AR1}}$) for comparable timescales. That is, there is always a $\phi_{\text{AR1}\;}$ value that represents the AR1 projection, even if the time series was generated by a more complex process. To define a feasible parameter range for simulation, we referred to the Human Connectome Project dataset, where ${\overset{ˆ}{\phi }}_{\text{AR1}\;}$ estimates ranged from +0.1 to +0.8. Accordingly, autocorrelation strength was varied using five positive $\phi_{\text{AR1}\;}$ values (0.1 to 0.8) with corresponding timescales $\tau_{\text{AR1}\;}$ from equation (7). This design resulted in a total of 15 simulation settings (three data-generating models × five autocorrelation strengths). For each setting, estimator performance was assessed by relative root mean squared error:

(44) $$rRMSE\left(\overset{ˆ}{\tau }\right)=\frac{\sqrt{\frac{1}{N}\sum_{n=1}^{N}{\left({\overset{ˆ}{\tau }}_{n}\;-\;\tau \right)}^{2}}}{\tau },\;rRMSE\left(\hat{se}\left(\overset{ˆ}{\tau }\right)\right)=\frac{\sqrt{\frac{1}{N}\sum_{n=1}^{N}{\left(\hat{se}\left({\overset{ˆ}{\tau }}_{n}\right)\;-\;se\left(\overset{ˆ}{\tau }\right)\right)}^{2}}}{se\left(\overset{ˆ}{\tau }\right)}.$$

#### 3.1.1 Time-Domain Simulations

In the *AR1 setting*, the data-generating process matches the fitted model, with time series simulated from an AR1 model:

(45) $$x_{t}=\phi x_{t-1}+e_{t},\;e_{t}\overset{iid}{∼}𝒩(0,\;1).$$

The *AR2 setting* introduces a mismatch between AR2 data-generating process and AR1 fitting. While AR2 allows for more complex dynamics (e.g., periodic signals), this study focused on stationary, aperiodic processes typical of rfMRI signals (He, 2011). As a result, the simulations were limited to stationary and aperiodic AR2 processes, with five pairs of AR2 coefficients selected so that the AR1 projections matched the above setting (see Figure 2 Panel A). The following model was used:

(46) $$x_{t}=\phi_{1}x_{t-1}+\phi_{2}x_{t-2}+e_{t},\;e_{t}\overset{iid}{∼}𝒩(0,\;1).$$

The *HCP setting* did not follow an autoregressive process, using instead empirical ACFs from five brain regions of subject #100610 from the HCP dataset (see Dataset Description). These regions were selected to match the $\phi_{\text{AR1}}$ projections above. To simulate time series with the same autocorrelation structure as the empirical data, we sampled from a multivariate normal distribution $𝒩(0,\overset{ˆ}{\Sigma })$, where $\overset{ˆ}{\Sigma }\in R^{K\times K}$ is the covariance matrix constructed from the sample ACFs. Under stationarity, the matrix $\overset{ˆ}{\Sigma }$ has a Toeplitz structure, meaning its *k*^th^ off-diagonal elements represent the sample ACF at lag $k:{\overset{ˆ}{\Sigma }}_{i-k,j-k}={\overset{ˆ}{\Sigma }}_{i+k,j+k}={\overset{ˆ}{\rho }}_{k}$. A Cholesky decomposition of the covariance matrix $\overset{ˆ}{\Sigma }=LL^{⊤}$, where L is a lower triangular matrix, was multiplied with Gaussian white noise:

(47) $$x_{t}=Le_{t},\;e_{t}\overset{iid}{∼}𝒩(0,\;1).$$

#### 3.1.2 Autocorrelation-Domain Simulations

Lastly, generating data in the time domain as described above creates an inherent disadvantage for the autocorrelation-domain method when evaluating estimator performance. This method requires a two-step estimation process: (1) computing the sample autocorrelation function (14) and (2) fitting the exponential decay function (16), resulting in cumulative estimation errors. To isolate and evaluate parameter recovery specific to step (2), we directly generated ACFs $\rho_{k}=\left\{\rho_{0},\rho_{1},\ldots ,\rho_{K}\right\}$ with K=4800 using the following signal + noise models:

(48) $$AR1:\rho_{k}=\phi^{k}+e_{k},$$

(49) $$AR2:\rho_{k}=\phi_{1}\rho_{k-1}+\phi_{2}\rho_{k-2}+e_{k},$$

(50) $$HCP:\rho_{k}={\overset{ˆ}{\rho }}_{k}+e_{k}.$$

Here, $e_{k}\overset{\;\text{iid}}{∼}𝒩(0,\;1)$ for all settings, and ${\overset{ˆ}{\rho }}_{k}$ represents the empirical autocorrelations obtained from subject #100610 from the HCP dataset. While this setting is unrealistic in practice when working with time series data, it enables us to decouple the estimation steps of the autocorrelation-domain method and perform a specific comparison with the single-step time-domain method.

### 3.2 Simulation Results

#### 3.2.1 Results for Autoregressive Simulations

**Figure 1:**
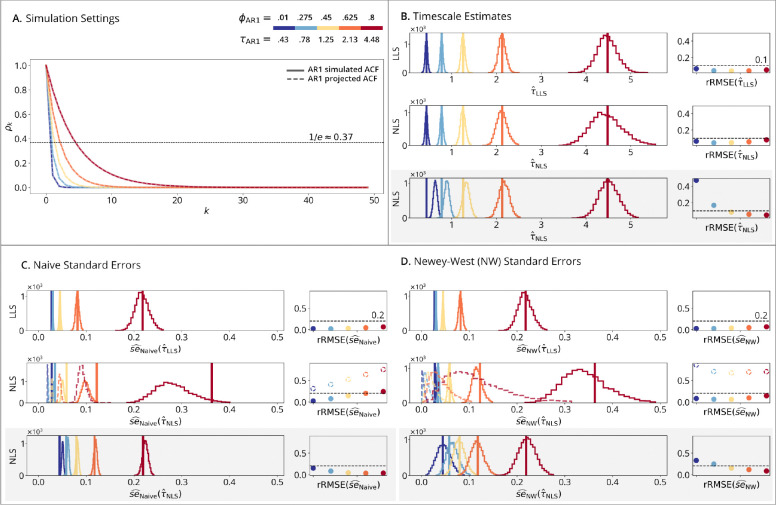
AR1 simulations. (**A**) Simulation Setting: solid lines show the simulated ACFs; dashed lines show the AR1-projected ACFs, which are overlapping as both follow AR1. Horizontal line marks the timescale where the AR1-projected ACF reaches 1/e≈0.37. (**B**) Timescale Estimates: vertical lines show true timescales; histograms show estimates across N=10,000 replications; points show rRMSE versus a 10% error line. (**Row 1**): LLS estimator fit to time-series data. (**Row 2**): NLS estimator fit to sample ACFs from time-series data. (**Row 3**): NLS estimator fit to theoretical ACFs with added noise, which is grayed out to indicate it is not a realistic setting. (**C**) Naive and (**D**) Newey-West Standard Errors: vertical lines show standard deviations from panel B; histograms show standard error estimates; points show rRMSE versus a 20% error line. (**Row 1**): time-domain standard errors fit to time-series data. (**Row 2** dashed lines): autocorrelation-domain standard errors fit to sample ACFs from time-series data. (**Row 2** solid lines): autocorrelation/time-domain standard errors fit to time-series data. (**Row 3**): autocorrelation-domain standard errors fit to theoretical ACFs with added noise.

AR1 simulations (Figure 1) are correctly specified because the data-generating process aligns with the fitted models, where each time series is generated from a AR1 process and each ACF by an exponential decay (**Panel A**). **Panels B-D** show how accurately the timescale estimators recover the true parameters and their standard errors as the timescale increases. **Panel B** shows that while larger timescales increase estimate variability, relative RMSE remains below 10%. However, at small timescales in the autocorrelation-domain simulations (**Panel B row 3**), NLS shows an upward bias when fit directly to noisy theoretical ACFs – a bias not present in the more realistic time-domain simulations. **Panels C-D row 1** shows minimal difference in naive versus Newey-West standard errors under correct specification, and both give accurate estimates. **Panels C-D row 2** (dashed lines) illustrate the Autocorrelation-Domain Method fit to sample ACFs from time-series data, showing that both naive and Newey-West standard errors are underestimated with high rRMSE. This occurs because the method incorrectly assumes a signal + noise ACF for an AR1 process. While the timescale estimates in **panel B row 2** remain unbiased due to the correct signal specification, the standard errors are biased toward zero because the model introduces variability that is absent in the true process. **Panels C-D row 2** (solid lines) show that this variability is present in the time domain, allowing the Autocorrelation/Time-Domain Method to uncover true standard errors, particularly for the Newey-West estimator with less than 20% error. Finally, **panels C-D row 3** show that when the data are generated from AR1 ACFs with added noise, then the Autocorrelation-Domain Method accurately estimates standard errors.

**Figure 2:**
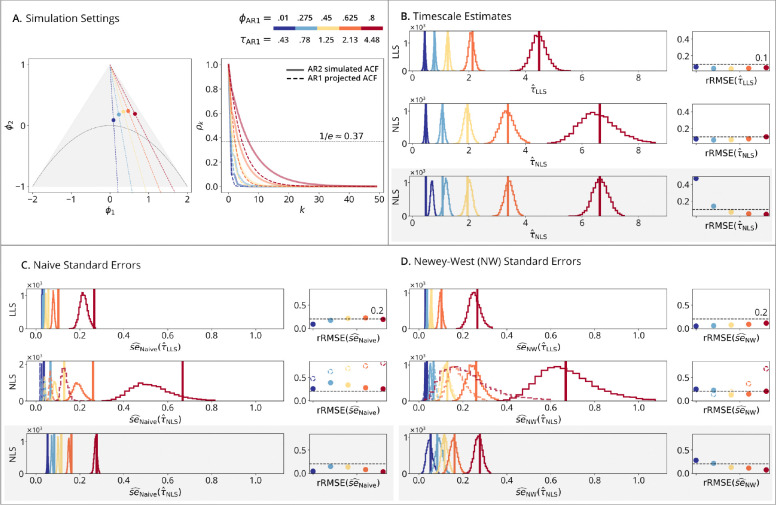
AR2 simulations. (**A**) Simulation Setting: triangle shows AR2 stationary region in the ($\phi_{1},\phi_{2}$) plane with a periodic/aperiodic boundary at $\phi_{2}=-\phi_{1}^{2}/4$. Points show five AR2 ($\phi_{1},\phi_{2}$) pairs with AR1 projections given by the colorbar. Solid lines show the simulated ACFs; dashed lines show the AR1-projected ACFs, which are not overlapping because the simulated AR2 is different from the fitted AR1. (**B**) Timescale Estimates: vertical lines show true timescales; histograms show estimates across N=10,000 replications; points show rRMSE versus a 10% error line. (**Row 1**): LLS estimator fit to time-series data. (**Row 2**): NLS estimator fit to sample ACFs from time-series data. (**Row 3**): NLS estimator fit to theoretical ACFs with added noise. (**C**) Naive and (**D**) Newey-West Standard Errors: vertical lines show standard deviations from panel B; histograms show standard error estimates; points show rRMSE versus a 20% error line. (**Row 1**): time-domain standard errors fit to time-series data. (**Row 2** dashed lines): autocorrelation-domain standard errors fit to sample ACFs from time-series data. (**Row 2** solid lines): autocorrelation/time-domain standard errors fit to time-series data. (**Row 3**): autocorrelation-domain standard errors fit to theoretical ACFs with added noise.

AR2 simulation results (Figure 2) explore how timescale estimators perform when the data-generating process is AR2, introducing misspecification since the estimators fit AR1 projections. In this setting, the goal is to assess the impact of specification error on timescale estimation. **Panel A** illustrates the AR2 ACF and its AR1 projection, emphasizing the mismatch between the true and fitted models. **Panel B** shows that the true timescale differs between the two estimators due to their respective definitions. Despite misspecification, both timescale estimators remain mostly unbiased (except that NLS shows an upward bias at small timescales in the autocorrelation-domain simulations, like the AR1 results). **Panel C** illustrates underestimation of standard errors by the naive estimator across all time- and autocorrelation-domain methods, where the downward bias is corrected by the Newey-West standard errors of **Panel D**. As with the AR1 results, **panels C-D row 2** (dashed lines) show that the Autocorrelation-Domain Method fit to sample ACFs from time-series data have high rRMSE and the true standard errors are unrecoverable at large timescales even with the Newey-West approach – another example that ACFs of AR processes cannot be represented as signal + noise models. **Panels D row 2** (solid lines) show that the Autocorrelation/Time-Domain Method is unbiased, as with **panel D row 3** for data generated from AR2 ACFs with added noise.

#### 3.2.2 Results for Realistic rfMRI Simulations

**Figure 3:**
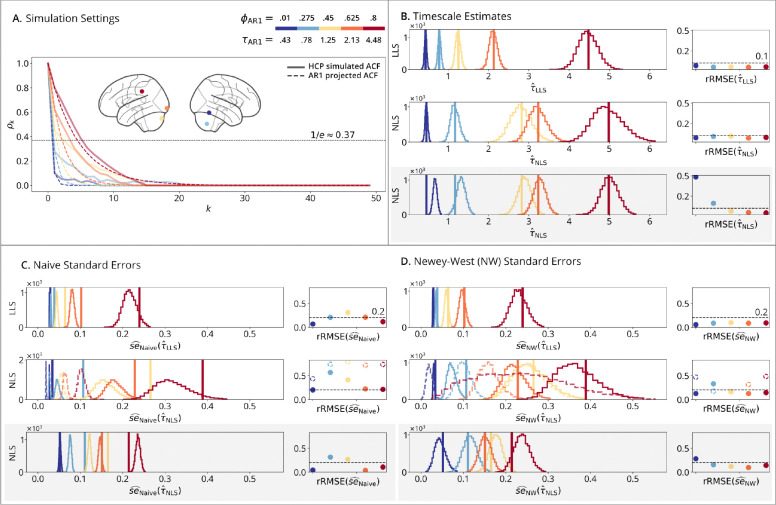
Realistic rfMRI simulations. (**A**) Simulation Setting: solid lines show simulated ACFs from five brain regions of HCP subject #100610; dashed lines show the AR1-projected ACFs, which do not overlap. (**B**) Timescale Estimates: vertical lines show true timescales; histograms show estimates across N=10,000 replications; points show rRMSE versus a 10% error line. (**Row 1**): LLS estimator fit to time-series data. (**Row 2**): NLS estimator fit to sample ACFs from time-series data. (**Row 3**): NLS estimator fit to theoretical ACFs with added noise. (**C**) Naive and (**D**) Newey-West Standard Errors: vertical lines show standard deviations from panel B; histograms show standard error estimates; points show rRMSE versus a 20% error line. (**Row 1**): time-domain standard errors fit to time-series data. (**Row 2** dashed lines): autocorrelation-domain standard errors fit to sample ACFs from time-series data. (**Row 2** solid lines): autocorrelation/time-domain standard errors fit to time-series data. (**Row 3**): autocorrelation-domain standard errors fit to theoretical ACFs with added noise.

Realistic rfMRI simulations (Figure 3) explore the performance of timescale estimators when simulating empirical processes derived from five distinct brain regions from a single subject. Like the AR2 results described above, this simulation method is designed to test estimator performance under model misspecification, as the data-generating process reflects realistic brain dynamics, while the fitted models project a simpler AR1 process. **Panel A** shows the mismatch between empirical ACFs and AR1 projections. **Panel B** presents the timescale estimates, where the LLS and NLS estimators yield different timescales due to their respective definitions. **Panels C-D** demonstrate that naive standard errors are underestimated; these errors are largely corrected by applying the Newey-West method. As with the AR simulations, the Autocorrelation-Domain Method approach is effective only when data are generated from ACFs with added noise. Otherwise, standard errors are only accurate when fit in the time domain, regardless of the domain used for timescale estimation. These results are consistent with the AR2 simulations using more realistic settings.

## 4 Data Analysis

### 4.1 Dataset Description

Resting fMRI (rfMRI) scans were provided by the Human Connectome Project (HCP), WU-Minn Consortium (led by principal investigators David Van Essen and Kamil Ugurbil; 1U54MH091657) funded by the 16 NIH Institutes and Centers supporting the NIH Blueprint for Neuroscience Research, and by the McDonnell Center for Systems Neuroscience at Washington University (Van Essen et al., 2013). Informed consent was obtained from all participants. Two subsets of the dataset were used: one for methods development and defining realistic simulation parameters (see Simulations), and the other for estimating high-resolution timescale maps of the cortex.

The *methods development subset* included 10 subjects (#100004 - #101410) scanned with a 3T gradient-echo EPI sequence (TR=720ms, slice thickness=2mm). Each subject completed four 15-minute runs (4800 timepoints total), preprocessed with standard steps including motion regression and artifact removal (see Glasser et al. (2013) for details). The resulting dataset dimensions were {10 subjects, 4800 timepoints, 300 regions}. The *timescale mapping subset* included 180 subjects scanned with a 7T gradient-echo EPI sequence (TR=1000ms, slice thickness=1.6mm) over four 16-minute runs (3600 timepoints total), using the same preprocessing steps. Functional data were analyzed on the cortical surface down-sampled to 2mm spatial resolution, yielding a dataset with the dimensions {180 subjects, 3600 timepoints, 64984 vertices}. The time- and autocorrelation-domain methods were fit to each vertex independently, a mass-univariate analysis approach that resulted in subject-level maps of timescale estimates and their standard errors.

#### 4.1.1 Group-level Analysis

Group-level maps combined individual timescales and standard errors, accounting for within- and between-subject variability. While remaining within the mass-univariate framework, for simplicity, we express the group timescale for the N=180 individual subjects at a single cortical vertex:

(51) $${\overset{ˆ}{\tau }}_{n}\;\text{for}\;n\in \{1,\;2,\ldots ,N\},$$

(52) $$\overset{‾}{\tau }=\frac{1}{N}\sum_{n=1}^{N}{\overset{ˆ}{\tau }}_{n}.$$

The group-level standard error for the timescale is given by the law of total variance:

(53) $$\hat{se}(\overset{‾}{\tau })=\sqrt{\frac{1}{N}\sum_{n=1}^{N}\hat{se}{\left({\overset{ˆ}{\tau }}_{n}\right)}^{2}+\frac{1}{N}\sum_{n=1}^{N}{\left({\overset{ˆ}{\tau }}_{n}-\overset{‾}{\tau }\right)}^{2}}.$$

Here, the first term under the square root is the within-individual variance and the second term is the between-individual variance.

For visualization, brain-wide t-statistic maps tested whether timescales exceeded 0.5 seconds ($H_{0}\;:\;\tau \;\leq \;0.5$) by the ratio:

(54) $$t\left(\overset{‾}{\tau }\right)=\frac{\overset{‾}{\tau }\;-\;0.5}{\hat{se}\left(\overset{‾}{\tau }\right)}.$$

Additionally, relative standard error (RSE) maps are presented to visualized the spatial precision of timescale estimates by the ratio:

(55) $$rse\left(\overset{‾}{\tau }\right)=\frac{\hat{se}\left(\overset{‾}{\tau }\right)}{\overset{‾}{\tau }}.$$

### 4.2 Data Analysis Results

#### 4.2.1 Results for rfMRI Timescale Maps

Subject-level maps were generated by mass-univariate fitting of LLS and NLS estimators to cortical surface data, where the NLS was fit by the two-step approach detailed in Autocorrelation/Time-Domain Method. **Panel A** presents the spatial distribution of timescale estimates, which show that NLS estimates tend to yield larger timescales than LLS, consistent with simulation results. **Panel B** shows the corresponding maps of standard errors, which appear align with the timescale maps, consistent with the simulation finding that larger timescales are associated with greater sampling variability. **Panel C** depicts the relative ratio of timescale estimates to their standard errors (i.e., t-statistics), testing whether the timescales significantly exceed a half second ($H_{0}:\tau \leq 0.5$). Despite larger standard errors for higher timescales, these regions still exhibit higher t-statistics. **Panel D** shows low RSEs across much of the brain indicating high estimation reliability.

Group-level timescale maps were generated by combining individual estimates to account for both within-individual and between-individual variability, providing an aggregate view of timescale distributions across subjects. **Panel A** shows that the average timescale maps for both LLS and NLS estimators are smoother than the individual maps, displaying a well-organized spatial pattern across the cortex, with NLS estimates generally being larger than LLS. **Panel B** presents the standard error maps, which combine variances from within-subject Newey-West estimates and between-subject timescale estimates. As expected from the simulation results, the standard errors are larger for NLS than for LLS. **Panel C** depicts t-statistics testing whether timescales exceed a half second, showing that both methods yield comparable results. **Panel D** plots relative standard errors (RSEs), illustrating the general trend that regions with larger timescales are easier to estimate, while areas with smaller timescales exhibit greater uncertainty. This is particularly apparent in the limbic network comprising orbital frontal cortex and anterior temporal cortex. **Panel E** highlights the spatial organization of timescales into networks, by mapping the t-statistic at each vertex to one of seven networks from the Thomas Yeo et al. (2011) atlas. The ordering {limbic, somatomotor, ventral attention, visual, dorsal attention, default, frontoparietal} aligns with the sensory-to-association axis of brain organization, and is consistent with a large literature on the hierarchical organization of timescales (Murray et al., 2014; Hasson et al., 2008; Stephens et al., 2013; Raut et al., 2020; Gao et al., 2020; Hasson et al., 2008). Sensory networks (limbic and somatomotor) have short timescales, followed by attentional networks (ventral and dorsal attention), and finally higher-order association networks (default mode and frontoparietal) contain the largest timescales.

This empirical analysis highlights methodological considerations for estimating fMRI timescale maps. (1) Both time-domain (LLS) and autocorrelation-domain (NLS) methods produce similar maps, but diverge at extremes – NLS yields larger timescales. The corresponding standard errors for NLS are also larger, so the resulting t-ratio maps remain similar between the two methods, highlighting why point estimates can be misleading without considering standard errors. Likewise, estimates are on average more precise for LLS, consistent with simulation results. (2) In this mass-univariate analysis, the computational cost of LLS is substantially lower than NLS because of its simple analytical solution. (3) The t-statistic maps organized by brain network exhibit a clear hierarchical timescale organization, reflecting how networks integrate and process information over time. And this pattern is consistent regardless of the estimation method. Taken together, these findings suggest that time-domain method may be preferable for large-scale neuroimaging studies due to its computational efficiency and higher precision, while producing maps consistent with previously reported timescale hierarchies.

## 5 Conclusions

This study introduces statistical methods for mapping fMRI timescales. We detail the large-sample properties of time- and autocorrelation-domain methods, showing both give estimates that converge, but to different values. This difference arises from the distinct definitions of the timescale parameter inherent to each method. We also demonstrate that both estimators yield consistent standard errors under broad conditions, allowing reliable inference and hypothesis testing. This addresses a major limitation in neural timescale studies that typically report only point estimates without uncertainty measures.

Simulation results highlight differences in finite sample bias and variance between the time- and autocorrelation-domain methods. All timescale estimators show largely unbiased results, with the linear least squares (LLS) method performing as well as or better than nonlinear least squares (NLS) in terms of relative root mean square error (rRMSE). Standard error estimates exhibit pronounced differences between naive and Newey-West corrected methods, particularly under misspecified settings such as AR(2) processes and realistic rfMRI data. Notably, standard errors cannot be directly estimated in the autocorrelation domain from sample autocorrelation functions (ACFs) of time-series data, necessitating a hybrid approach as detailed in Autocorrelation/Time-Domain Method. Furthermore, time-domain methods demonstrate comparable or superior performance to autocorrelation-domain methods across all simulation scenarios. Specifically, the time-domain linear method (3) offers greater computational efficiency and estimation stability for smaller timescales, whereas the autocorrelation-domain nonlinear method (8) accommodates longer-range autocorrelations at the cost of reduced accuracy. The application of Newey-West corrected standard errors enhances inference reliability by reducing bias across both methods (Newey and West, 1987), particularly evident in scenarios where autocorrelation decay is not exponential.

Applied to HCP rfMRI data, both methods yield timescale and t-statistic maps consistent with known functional hierarchies (Van Essen et al., 2013). These results align with prior work demonstrating larger timescales in associative versus sensory cortices (Raut et al., 2020; Shafiei et al., 2020; Lurie et al., 2024; Mitra et al., 2014; Kaneoke et al., 2012; Wengler et al., 2020; Shinn et al., 2023; Manea et al., 2022; Ito et al., 2020; Müller et al., 2020). The spatial patterns reinforce the hypothesis that hierarchical organization governs temporal processing across cortical regions. However, mechanistic interpretations of these timescales – particularly their relationship to underlying neurophysiological processes – remain to be fully explored in future work.

Although rfMRI provides high spatial resolution compared to EEG, MEG, or ECoG, its sparse temporal sampling can introduce finite sample variability. Asymptotic properties (see Estimator Properties) assume large samples, but in practice, low-frequency sampling of strongly dependent hemodynamic processes can yield too small an effective sample size (Afyouni et al., 2019; Kaneoke et al., 2012). Moreover, the mixed metabolic and neuronal origins of the hemodynamic signal complicate mechanistic interpretations (Raut et al., 2020; He, 2011). Methodologically, deviations from stationarity and mixing can still affect reliability, despite our methods handling common forms of misspecification (Hansen, 2022, Chapter 14.7). Extending model definitions to account for nonstationarity, where autocorrelations are time-dependent, might provide more accurate maps, especially in task-based or dynamic fMRI paradigms (He, 2011). Additionally, adding standard errors to the frequency-domain approach to timescale estimation would allow for the direct modeling of oscillations, which is important when working with electrophysiological recordings of the brain (Donoghue et al., 2020; Gao et al., 2020).

In conclusion, we introduce robust rfMRI estimators for timescales and standard errors, enabling rigorous statistical comparisons across regions, conditions, and subjects. This advances the accuracy and interpretability of neural timescale maps. Our methods move beyond point estimates by incorporating variability for inference and testing. The work lays the methodological foundation for future research on the role of timescales in brain structure and function.

## Figures and Tables

**Figure 4:**
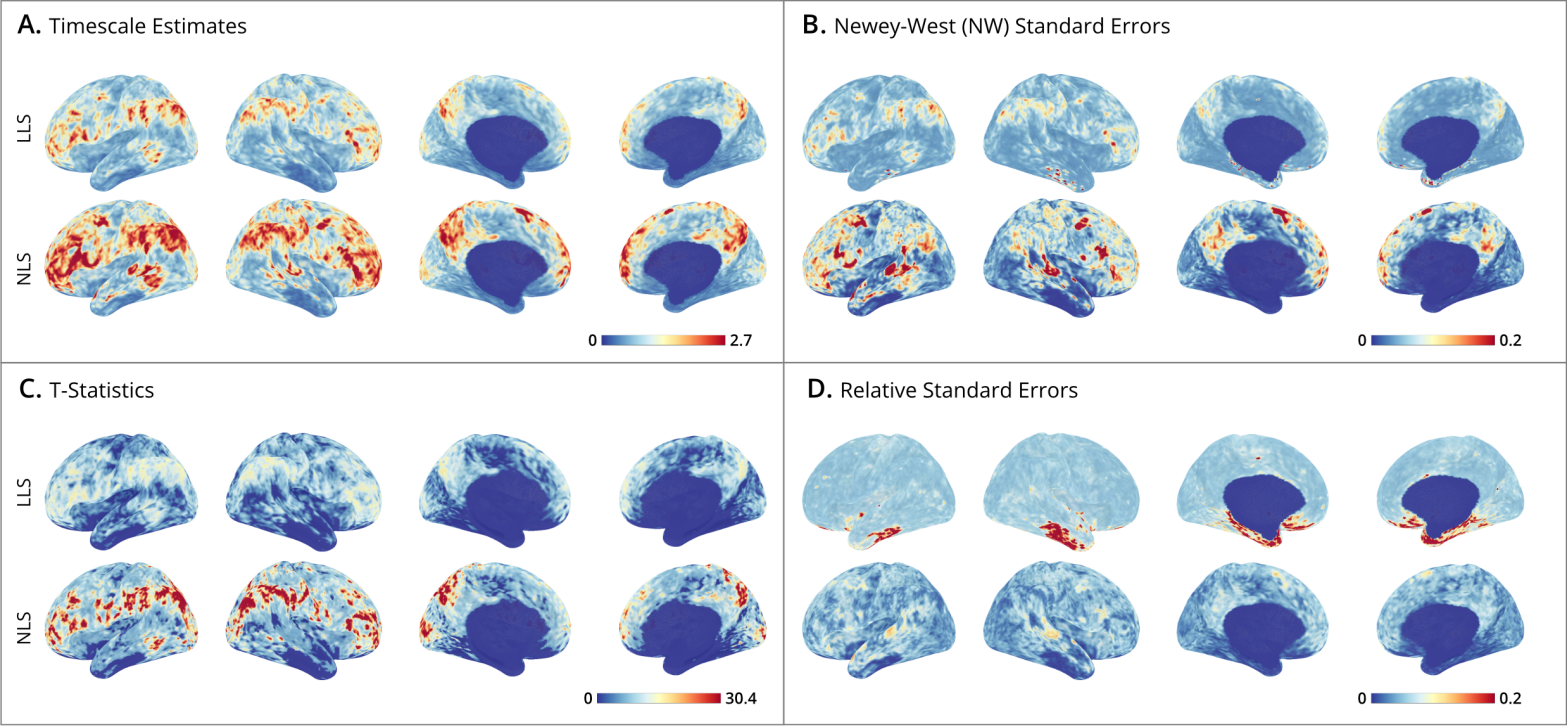
Human Connectome Project subject-level timescale maps. (**A-D**) Cortical surface maps from HCP subject #100610. Displays show lateral-left, lateral-right, medial-left, and medial-right views, plotted using the *StatBrainz* package (Davenport, 2025). The upper bounds on the colorbars are set for each panel at the 99^th^ percentile of cortical map values. For each panel, the top row shows results of the time-domain method (LLS), and the bottom row the autocorrelation-domain method (NLS). (**A**) Timescale estimates: maps display the timescales (in seconds) estimated at each vertex. (**B**) Newey-West standard errors: shows the spatial distribution of standard errors, where smaller values indicate greater estimation precision. (**B row 2**) shows the hybrid autocorrelation/time method. (**C**) T-statistics: unthresholded and uncorrected t-ratios testing where timescales exceed 0.5 seconds. (**D**) Relative Standard Errors (RSEs): relative reliability of estimates, where low RSE (near zero) indicates high precision with small uncertainty.

**Figure 5:**
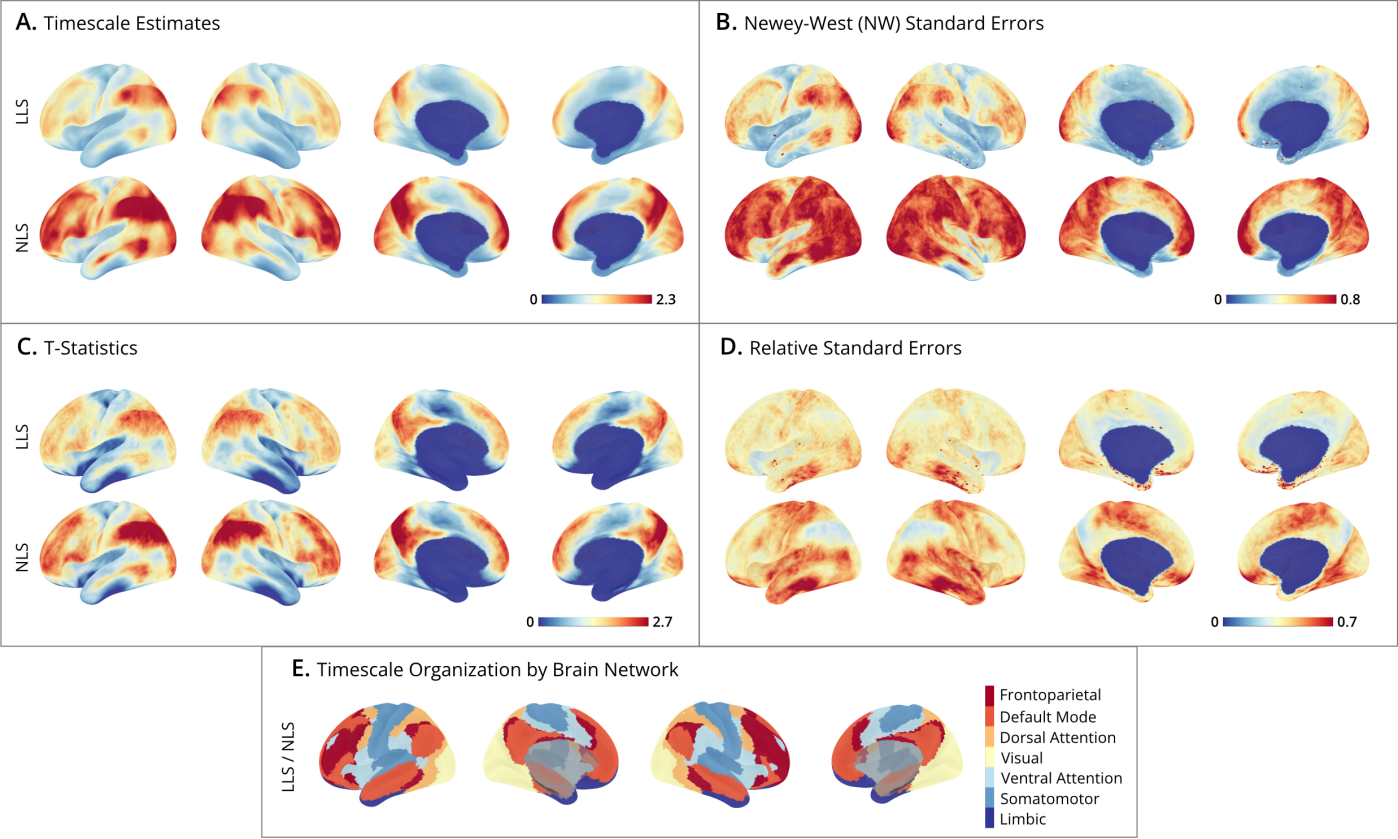
Human Connectome Project group-level timescale maps. (**A-D**) Cortical surface maps from N=180 HCP subjects. Displays show lateral-left, lateral-right, medial-left, and medial-right views, plotted using the *StatBrainz* package (Davenport, 2025). The upper bounds on the colorbars are set for each panel at the 99^th^ percentile of cortical map values. For each panel, the top row shows results of the time-domain method (LLS), and the bottom row the autocorrelation-domain method (NLS). (**A**) Timescale estimates: maps display the group-level timescales (in seconds) at each vertex, averaged over subjects. (**B**) Newey-West standard errors: group-level spatial distribution of estimates, accounting for within- and between-subject variability. Smaller values indicate greater precision. (**C**) T-statistics: unthresholded and uncorrected t-ratios testing where group-level timescales exceed 0.5 seconds. (**D**) Relative standard errors (RSEs): relative reliability of estimates, where low RSE (near zero) indicates high precision with small uncertainty across subjects. (**E**) Timescale organization by brain network: maps display brain networks from the Yeo 7 Network Atlas, ordered by the network-averaged t-statistics (from panel C). This ordering is the same for LLS and NLS methods, and highlights the hierarchical organization of timescales, progressing from sensory networks (e.g., somatomotor and limbic in blues) to association networks (e.g., frontoparietal and default mode in reds).

## Data Availability

All simulation results and fMRI timescale maps, inclusive of the code by which they were derived, can be accessed on github.com/griegner/fmri-timescales. The code is under the open source MIT license, allowing access and reuse with attribution. The Human Connectome Project young adult dataset (ages 22–35; 2018 release) used in this study is publicly accessible under a data usage agreement, which describes specific terms for data use and sharing.
